# Characterizing Navigational Changes in Preclinical Alzheimer’s Disease: A Route Complexity Metric Derived From Naturalistic Driving Data

**DOI:** 10.1109/JTEHM.2025.3619802

**Published:** 2025-10-09

**Authors:** Kelly Long, Ganesh M. Babulal, Sayeh Bayat

**Affiliations:** Department of Biomedical EngineeringUniversity of Calgary 2129 Calgary AB T2N 1N4 Canada; Department of NeurologyWashington University School of Medicine 7284 St. Louis MO 63110 USA; Institute of Public Health, Washington University in St. Louis 7284 St. Louis MO 63110 USA; Department of Geomatics EngineeringUniversity of Calgary 2129 Calgary AB T2N 1N4 Canada; Hotchkiss Brain Institute, University of Calgary 2129 Calgary AB T2N 1N4 Canada

**Keywords:** Alzheimer’s disease, cognitive impairment, human mobility, navigational behaviour, driving, digital biomarkers, route complexity, GPS trajectory analysis, naturalistic data

## Abstract

Objective: To examine how early pathophysiological changes in Alzheimer’s disease (AD) affect navigational decision-making by analyzing the complexity of driving routes in older adults with and without preclinical AD. Methods: We developed a novel route complexity metric based on the number of left and right turns and the deviation from the most direct path, accounting for cognitive load during navigation. Naturalistic GPS driving data were collected for a year from 111 older adults aged 65–85, with preclinical AD status determined via cerebrospinal fluid amyloid biomarkers. A multiple linear regression model was used to assess the relationship between age, preclinical AD status, and route complexity. Results: The findings of this study indicate that preclinical AD may influence the navigational abilities of older adults. After controlling for age, participants with preclinical AD chose routes with higher baseline complexity than the control group. It further revealed that participants with preclinical AD selected routes with lower complexity as they aged—a trend not observed in healthy controls. Conclusion: Preclinical AD is associated with changes in spatial decision-making that are observable in real-world driving behaviours. The age-related decline in route complexity among those with preclinical AD may reflect compensatory strategies or progressive cognitive changes. Clinical Impact: This study presents a non-invasive, behaviour-based metric that could support early detection of cognitive decline. It may also inform the design of personalized mobility interventions and dementia-friendly mobility systems.

## I. Introduction

Alzheimer’s disease (AD) is a progressive neurodegenerative disease characterized by a decline in cognitive abilities, particularly memory, executive function and decision-making [1]. It is estimated that more than 55 million individuals worldwide are living with dementia, of which 60%-70% are caused by AD [1]. While memory impairment is one of the most recognized symptoms of AD, early-stage AD can also affect everyday behaviours, including how individuals choose to move through their environment [2], [3], [4], [5].

Long before cognitive symptoms become detectable in a clinical setting, there are pathological changes, such as the abnormal accumulation of beta-amyloid ( $A \beta $) and tau proteins that can be detected in the brain [6], [7]. These proteins interfere with inter-neuron communications, and are associated with subtle changes in cognition and behaviour [8]. This biomarker-defined stage, known as preclinical AD, presents an opportunity for identifying early behavioural markers of cognitive decline [9], [10]. Early detection during this stage is critical for enabling timely initiation of disease-modifying therapies and lifestyle interventions that may delay disease progression and improve patient outcomes. Translating early markers into clinical frameworks could additionally allow for personalized monitoring of at-risk individuals in real-world healthcare settings.

Driving is a cognitively complex activity [11], and is also an important aspect of the lives of many older adults. It is beneficial for maintaining independence and quality of life, and for many, driving can be linked to identity [12]. This makes it a logical area of study for those with preclinical AD. Previous research has shown that changes in driving behaviours, such as the number of jerks, driving at night, and changes in speed variability, can be observed in individuals with preclinical AD [3], [13], [14], [15]. These findings suggest that driving behaviour may serve as an indicator of early AD-related changes. Many of these driving behaviour changes can be measured non-invasively and continuously, providing scalable data for integration into digital health monitoring systems.

Previous studies on driving and preclinical AD have primarily focused on driving performance metrics, with less attention to route selection. However, there is growing evidence that navigation deficits—especially those related to allocentric navigation, which involves using landmarks and the surrounding environment to navigate—may influence the types of routes people choose to take [3], [16], [17]. As this ability declines, individuals may find it harder to plan or follow more complex routes [16], particularly those that require many turns or remembering spatial relationships. For example, people with preclinical AD or mild cognitive impairment have been shown to struggle with learning and recalling new routes in virtual mazes or real-world settings [18], [19], and often rely more on body-based (egocentric) strategies as the disease progresses [20]. Other studies have shown a relationship between AD biomarkers and a lower self-reported sense of direction, and that those with lower self-reported sense of navigation were also found to drive less often [5], [21]. These findings suggest that early changes in cognition due to AD may lead to changes in navigational behaviours, potentially leading people to adjust their route planning and execution.

In this study, we propose a novel route complexity metric that considers the cognitive load of a driving route using two key components: the quantity of left and right turns taken, and the deviation from the direct path. Our complexity metric was intentionally restricted to intrinsic route geometry (turn frequency and straightness) to isolate a behavioral signal of navigational strategy that is reproducible from GPS alone. Other factors such as traffic volume, road type, and environmental variability were not included in this first version, as they represent complementary dimensions of complexity that require further investigation. Using naturalistic GPS data collected from older adults with and without biomarker-confirmed preclinical AD, we examine whether route complexity differs across these groups, and how it relates to age.

Understanding the differences in the complexity of route choices between people with preclinical AD and those with normal aging can help us understand the real-world impacts of pathological changes on navigational and spatial decision-making, contributing to the development of non-invasive, behaviour-based tools for early detection of cognitive change. Such tools could supplement existing clinical assessments to provide context that could improve the accuracy and certainty of AD diagnoses. When comparing clinical and postmortem diagnoses, there is a 12-23% discrepancy [22]. To highlight the importance of this, only 20% of primary care providers surveyed in a 2019 study [23] were highly confident in interpreting results of cognitive tests, and even less (14%) reported high confidence in interpreting brain imaging findings.

## II. Methods

### A. Data Collection

Naturalistic driving data were collected using a GPS data logger (Azuga G2 Tracking Device TM) as part of an ongoing study conducted in the DRIVES Project at the Washington University School of Medicine [13], [24], [25]. The data logger was installed into the onboard diagnostics-II port of each participant’s vehicle, and data collected over time included the date, latitude, longitude, and speed data at 30-second intervals every time a trip was made. We only included trips that were entirely in the greater St. Louis area in 2019 to control for environmental and seasonal variability. We excluded a single outlier trip exceeding 14 hours, assuming that refueling would be necessary. Additionally, very short trips (under 0.02 mi) were excluded as these likely represented idling rather than actual driving. The study was approved by the Conjoint Health Research Ethics Board, University of Calgary (REB22-1005), and Washington University in St. Louis Institutional Review Board (IRB ID# 202010214 and 201706043). Informed consent was obtained from all participants, and research was performed in accordance with the tri-council policy statement on ethical conduct for research involving humans (TCPS 2, 2022).

Participants were required to be cognitively normal based on the Clinical Dementia Rating® (CDR) of 0 [26], have a valid driver’s license, drive at least once a week, and be willing to take part in biomarker collection. We included participants between ages 65 and 85 as those over 85 are often subject to more considerable clinical variability, such as the presentation of comorbidities or non-Alzheimer’s related cognitive and psychological changes [27], [28]. Non-English speakers were excluded due to potential communication barriers in providing informed consent. Participants took part in annual office testing, including but not limited to CDR, the Driving Habits Questionnaire [29], and checking the participant’s driver’s license to ensure they met the study requirements. A cadre of imaging and biofluid biomarkers were collected every two to three years. Preclinical AD status (yes/no) was based on biofluid biomarkers using established cut-offs from published studies ( $A \beta _{42}$/ $A \beta _{40} < $ 0.0673) [10], [30]. Participants with changes in preclinical AD status over the course of the study were excluded from the analysis.

### B. Preprocessing

Due to the sparse GPS sample rate, the driving trajectory data was snapped to a simplified road network, provided by Open Street Maps [31] using a map-matching process [32]. More detail is found in Appendix. Turns were identified by computing the heading angles ( $\theta $) of adjacent road segments using (1) where $\phi $ is longitude and $\lambda $ is latitude. If the heading of the second road segment minus that of the first road segment relative to the direction of travel was within 30 and 150 degrees, the turn was classified as a left turn. Similarly, if the difference between the second segment’s heading and the first segment’s heading falls between -150 degrees and -30 degrees, it was considered a right turn.

\begin{equation*} tan(\theta)=\cfrac {sin(\Delta \lambda)cos(\phi _{2})}{cos(\phi _{1})sin(\phi _{2})-sin{(\phi _{1})cos(\phi _{2})cos(\Delta \lambda)}}. \tag {1}\end{equation*}

**FIGURE 1.**
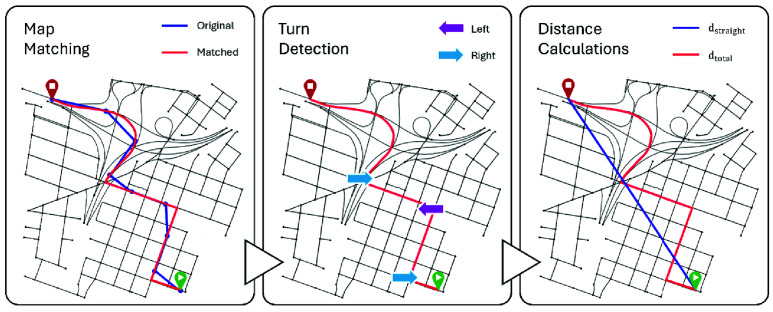
Preprocessing steps for raw trajectory data collected for the case study before computing complexity include map matching, turn detection, and distance calculations. The first step was map matching (left). The data was collected at a sample rate of 30s, which does not represent the actual route taken on the path; this is represented in the figure for map matching with the blue line, where the blue points correspond to the observations. The red line shows the route after snapping the route to the road network (map matching). The next step was turn detection (centre) on the map-matched trajectory. Finally, distances were calculated (right) from the matched trajectory to plug into the proposed complexity metric. The straight-line distance was computed between the start and end (blue), and the total distance was calculated as the length of the map-matched path (red).

The total route distance was computed as the sum of haversine distances between all the adjacent points, and the straight distance was computed as the haversine distance between the first and last observed positions on the route. The overall preprocessing pipeline is visualized in Fig. 1.

### C. Complexity Measure

Sometimes, routes are selected to minimize turns or to follow the simplest or most direct path to the destination [33]. Based on this principle, we quantified route complexity by integrating both geometric characteristics and the relative cognitive effort required to navigate a path. The metric accounts for cognitive demand associated with following an indirect route by incorporating both the deviation from a direct path and the additional load imposed by turns. In a clinical context, the cognitive load required could impact the decisions individuals make about which types of routes to follow, which may shift as cognitive function changes.

Based on previous research, which has shown that driving on curved paths is more cognitively demanding than driving on straight paths [34], [35], we assumed that less direct routes were more complex than straight-line routes. Furthermore, a recent study by Wisch et al. [36] showed that older adults who can quickly react to their surroundings (those with strong VAN x VAN and SAL x DAN brain network connections) tend to take straighter routes. In this context, a straightness value of one represents the most direct route, whereas smaller values indicate less direct routes. Since more indirect routes require more frequent adjustments and decision-making, we incorporated the inverse of straightness into our complexity metric to reflect the added cognitive burden of following less direct paths. This is represented by the ratio of the total on-road trip distance ( $d_{total}$) to the straight-line distance ( $d_{straight}$) between the origin and destination. This term also helps to characterize curves and turns as a larger ratio, which is indicative of more deviation from a straight line, leading to higher complexity values when curves and turns are present.

The complexity metric also includes a weighted turn term consisting of the number of left turns in the route (*l*) and the number of right turns (*r*). The weight parameter (*w*) is a value from zero to one that changes the relative effect of left turns and right turns on complexity. For example, when *w* is set to 0.5, it signifies that left and right turns are given equal consideration in contributing to the route’s complexity. When combined with the inverse straightness term, the equation assigns a higher complexity weight to turns than curves, as turns require more attention due to traffic considerations. A constant value of one was added to the weighted turn term to ensure that curves and windy roads would still be considered in the calculated value if there were no turns. The route complexity (*c*) was computed as follows in (2).

\begin{equation*}c=log \left [{{(wl+(1-w)r+1) \times \cfrac {d_{total}}{d_{straight}}}}\right ]. \tag {2}\end{equation*}

The assignment of a higher weight to left turns for driven routes in the proposed metric is recommended based on the empirical evidence highlighting their increased complexity. Specifically, a 2013 study [37] used a 3.0 Tesla MRI system (Magnetom TIM Trio software version b15, Siemens, Erlangen, Germany) to measure brain activity during simulated driving scenarios. The study found that left turns induced significantly more activation in the brain’s posterior regions than right turns, indicating a higher cognitive load. Another study found a significant increase in mental workload through decreased eye-blink frequency while turning (0.31 blinks per second for left turns and 0.29 blinks per second for right turns) compared to driving on a straight-away (0.376 blinks per second) [34]. The same study also found that response time to a secondary task was higher for both left turns (2.01 s) and right turns (1.84 s), compared to straight lane driving (1.48 s). These findings indicate slightly higher difficulty for left turns in a vehicle driving on the right side of the road.

In this study, we assigned a higher weight of 0.66 for left turns to reflect driving in an American metropolitan area (driving on the right side of the road with traffic considerations). This value was informed by practical driving experience, as well as supporting evidence from cognitive load studies described above, and crash statistics. From a driver’s perspective, left turns generally involve more simultaneous demands than right turns. For example, at a T-intersection without traffic lights, a left turn requires monitoring vehicles approaching from both directions on the main road, evaluating gaps in traffic, and checking for pedestrians. Visibility may also be more limited depending on the road design as the driver must turn farther across the intersection. In contract, a right turn at the same intersection typically only requires scanning for pedestrians and finding a gap in the nearest lane, illustrating how left turns create more attentional demands.

**FIGURE 2.**
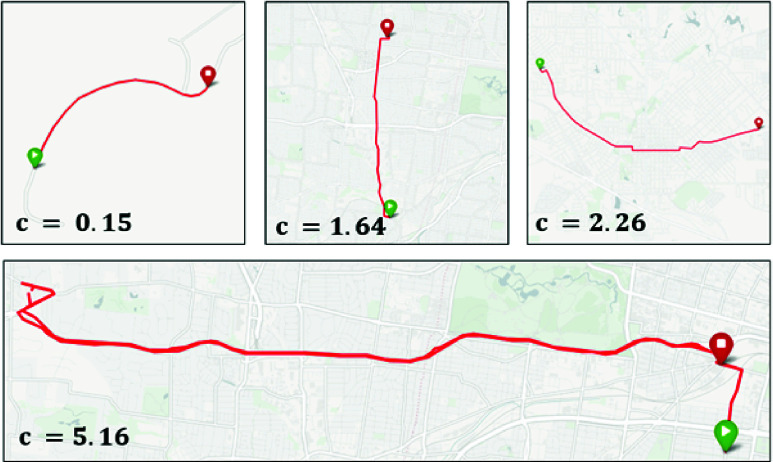
Visualization of three routes with varying complexities driven in St. Louis, Missouri. The travelled path is depicted by a red line, with the start of the route identified by a green pin and the end of the route identified by a red pin. A complexity value (c) was computed with the proposed metric and a weight of 0.66 and is overlayed on the map showing the corresponding route.

A study on crash factors quantified pre-crash events with 22.2% of crashes involving left turns compared to only 1.2% involving right turns [38].This highlights the disproportionate risk associated with left turns; however, risk is not synonymous with cognitive load. If the weighting were based only on response time differences [34], left turns would appear to be about 1.5 times more demanding than right turns (corresponding to a weight of 0.6), when each is compared to straight driving. However, response time is not a direct quantification of cognitive load, and it does not capture the attention demands of turning in naturalistic driving conditions. For these reasons, the weight of 0.66 was selected heuristically: it is higher than response times alone would imply, acknowledging crash risk, but closer to the cognitive evidence, which is more directly tied to workload. This choice is also consistent with real-world driving experience, where left turns are broadly recognized as more demanding than right turns. Fig. 2 shows routes of various complexities alongside their corresponding complexity values. The visualization shows that the equation assigns higher complexity values to routes with greater numbers of turns and less direct paths, whereas more direct routes with fewer turns are assigned lower complexity values.

**FIGURE 3.**
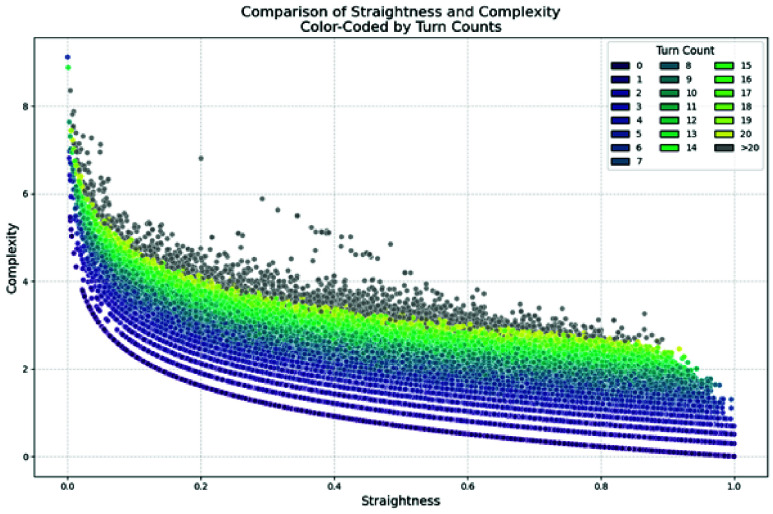
The relationship between complexity and route straightness, coloured by the number of turns.

Fig. 3 shows the relationship between complexity and straightness, with points coloured by the total number of turns in each trip. The visualization demonstrates that complexity is inversely correlated to straightness, as expected, given inverse of straightness is a factor in its calculation. Additionally, the coloured points indicated that an increased number of turns contributes to a higher complexity value. The colours do not correspond to a linear increase complexity, highlighting the effect of the weighted turn term in the complexity equation.

### D. Statistical Analysis

We used (2) to calculate the complexity of each driving trip over one year of data, beginning in January 2019, for each participant, applying a weight of $w =0.66$. Round trips were excluded from further analysis as such routes would result in an infinitely high complexity value. This limitation arises because the metric measures the complexity of a route in terms of getting between the origin and destination, and round trips inherently lack a clear origin-to-destination path.

To ensure coverage of all seasons, we took the average value for each participants. We then conducted a multiple linear regression analysis using the Statsmodels library [39] to model the mean complexity as a function of age for individuals with and without preclinical AD. To assess whether the relationship between complexity and age varied between healthy controls and individuals with preclinical AD, we included an interaction term in the model. Additionally, we estimated secondary model s incorporating sex and race as covariate s to evaluate their potential influence. However, neither the sex term nor the race term were statistically significant and they did not affect the overall model results, indicating they were not necessary adjustment variable s in this analysis.

## III. Results

Table 1 describes the demographics of the sample population, with statistical tests conducted to compare the control and preclinical AD groups: chi-squared contingency tests were used for categorical variables and t-tests for continuous variables (all normally distributed with equal variance). Approximately 37% of the sample had preclinical AD, both groups with average ages in the mid-70s. Participants with preclinical AD were older on average by about one year (p <0.05). The sample was split relatively evenly between male and female, with close to 50% of the participants being male and the demographic was primarily non-Hispanic white but included a small sample of African American participants, with no significant group differences in sex or race between the control and preclinical AD groups. Each group drove multiple times a day on average with a large standard deviation in the number of trips. The control group drove more often on average; but this difference was not statistically significant. Additionally, we examined whether driving complexity differed between the control and preclinical AD groups and found no significant difference when not accounting for age or other variables.

**TABLE 1:** Participant Demographics With Results of T-Tests (Continuous Variables) and Chi-Squared Tests (Categorical Variables)

	Control (n=70)	Preclinical AD (n=41)	Test	p-value
Age± s.d. [years]	$73.7~\pm ~3.7$	$74.8~\pm ~3.8$	*t* = -2.363	< 0.05
Sex [% male]	55.7	48.8	$\chi ^{2}$ = 0.499	0.480
Race [% African American]	18.6	7.3	$\chi ^{2}$ = 2.655	0.103
Trips ± s.d.	$1168.7~\pm ~678.8$	$1070.7~\pm ~592.4$	*t* = 0.768	0.444
Complexity ± s.d.	$1.63~\pm ~0.21$	$1.59~\pm ~0.21$	*t* = 1.040	0.301

**FIGURE 4.**
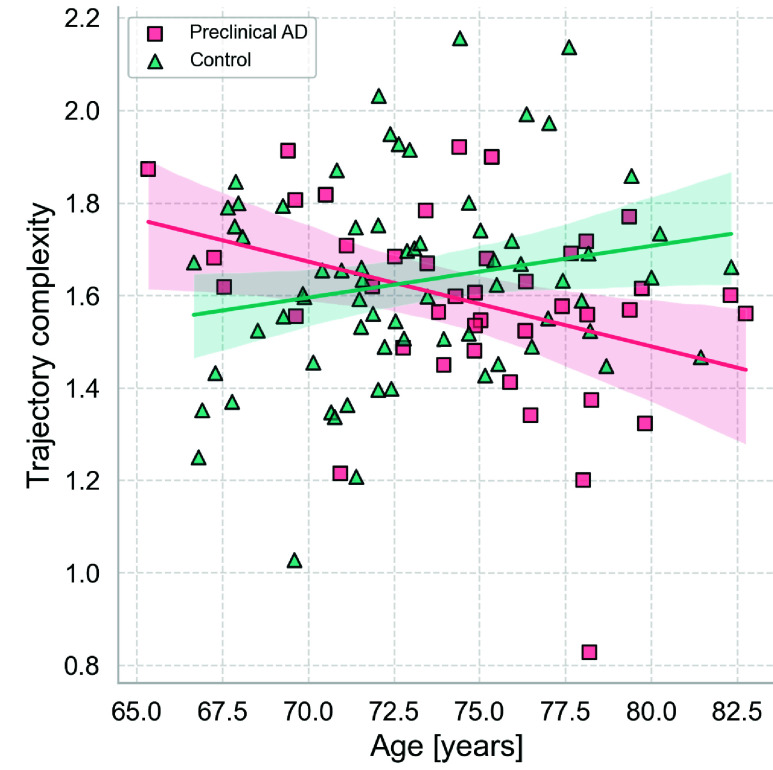
Fitted multiple linear regression of the mean complexity (y) of driving routes over the study period as a function of age (x) for participants with (pink) and without (cyan) preclinical AD. The linear model shows a significant interaction effect with a reduction in mean complexity with age for participants with preclinical AD.

The assumptions for multiple linear regression were met. We tested the normality assumption with a QQ plot, checked for homoscedasticity by observing that the residuals were scattered randomly around zero, and verified the independence of observations. Table 2 summarizes the results of the multiple linear regression. The results suggest that while there is no significant association of complexity with age (near significant), complexity significantly differed in participants with and without preclinical AD after accounting for age; specifically, the presence of preclinical AD biomarkers increased the intercept by 2.145 (p < 0.05). Furthermore, there was a statistically significant interaction effect between age and preclinical AD for older adults between ages 65 and 85. The negative coefficient implies that driving route complexity decreased with age for participants with preclinical AD (p < 0.05), whereas there is no significant change for healthy controls. This effect is visualized in Fig. 4. The model explains only 8% of the variation in the complexity data ( ${\mathrm {R}}^{2} =0.079$); however, the overall model’s F-statistic is significant to the 95% confidence level (F = 3.064, p < 0.05), suggesting that the model is a good fit for the data.

**TABLE 2:** Multiple Linear Regression Showing a Significant Age-by-Preclinical AD Interaction Effect on Route Complexity

Coefficients	Estimate	Standard Error	t-value	p-value
Intercept	0.814	0.475	1.715	< 0.1
Age	0.011	0.067	1.716	< 0.1
Preclinical AD (Yes)	2.145	0.772	2.777	< 0.05
Age $\times $ Preclinical AD (Yes)	-0.030	0.010	-2.837	< 0.05

## IV. Discussion

### A. Interpretation of Results

Among cognitively normal participants, though statistically insignificant (p < 0.1), Fig. 4 suggests a slight trend toward higher route complexity with age. This trend might reflect previous research that shows that increasing age in healthy older adults was associated with higher self-regulation of driving [40], [41], [42]. Specifically, they may avoid highways and instead take local or familiar roads. Such routes typically involve a higher number of turns and less linearity, which, according to our complexity measure, would correspond to greater route complexity.

The findings of this study indicate that preclinical AD may influence the navigational abilities of older adults. After controlling for age, participants with preclinical AD chose routes with higher baseline complexity than the control group. Considering the comparative analysis in Table 1 was insignificant for the complexity metric, the significant cross-sectional effect for preclinical AD status, suggests that the differences in route complexity are related to the interplay between age and preclinical AD. The analysis further revealed that participants with preclinical AD selected routes with lower complexity for driving as they aged, a trend not observed in healthy controls.

One possible interpretation is that biomarker-positive adults may initially engage in less self-regulation, but may gradually shift towards simpler routes, suggesting that preclinical AD could impair cognitive functions related to planning and decision-making more significantly with higher age, leading to simpler driving routes over time. This shift aligns with the notion of compensatory behaviour, where individuals with subtle cognitive changes adapt their mobility decisions to maintain safety and independence. In a clinical setting, these patterns may contribute to non-invasive behavioural markers for preclinical AD. With more research it could also complement cognitive testing in the earlier clinical stages of AD and contribute to continuous monitoring of the disease progression.

### B. Clinical Translation

The route complexity metric employed in this study offers a novel perspective on spatial cognition and human mobility. The use of this metric could be of particular interest in populations affected by neurodegenerative conditions such as Alzheimer’s disease or traumatic brain injuries [2], [3], [4], [5], [43] that can impact navigational abilities and spatial thinking. Furthermore, longitudinal analysis could facilitate tracking behavioural changes over time and provide insights into how decision-making processes evolve over time in response to cognitive decline.

Looking ahead, combining the proposed complexity metric with other behavioural indicators of mobility and with established fluid or imaging biomarkers could enable the creation of an integrated framework for understanding mobility and cognition. Such integrative approaches may improve diagnostic confidence, allow more sensitive tracking of early decline, and support personalized intervention planning. They may also guide the development of dementia-friendly mobility systems; for example, through navigation aids that simplify route options or emphasize less complex paths.

### C. Limitations and Future Directions

Despite its utility, the proposed complexity measure has some limitations that should be acknowledged. These limitations relate to the scope of the metric, data collection and processing, as well as interpretation. Together, these limitations highlight areas for refinement and future work.

#### 1) Traffic and Environmental Considerations

The proposed measure does not incorporate local road network attributes or environmental factors that may influence route selection—such as traffic signals, stop signs and the availability of alternative routes. As a result, it may introduce bias by overlooking key contributors to cognitive load during real-world navigation. Importantly, this metric is distinct from traditional wayfinding measures, which are typically concerned with an individual’s ability to navigate through an environment using spatial cues, landmark recognition, and spatial awareness. In contrast, the proposed complexity measure captures the structural characteristics of a route, serving as an indirect marker of cognitive load. This distinction is important for clinical translation, as it highlights that the measure is not a diagnostic tool in isolation, but it can be a scalable, real-world behavioural signal, complementary to other assessments.

On a similar note, traffic can represent a complementary dimension of complexity. Traffic volume and road capacity may also influence how complex a route feels. In the United States, traffic volume data is routinely collected and most roads have Average Annual Daily Traffic (AADT) available, and roads are assigned a National Functional Class (NFC) describing the scale or purpose of the road (arterial, collector, local) [44]. These attributes may help explain route choices, as some drivers avoid higher-capacity or higher-class roads due to stress or safety concerns [45]. Despite this potential, several challenges arise. AADT and NFC are broad measures that cannot capture temporal variation.

Traffic and the proposed route complexity metric represent different but complementary dimensions of complexity. The metric developed in this study reflects structural parameters of the chosen route that proxy cognitive load, while traffic reflects contextual conditions that can also shape driver decisions and perceived difficulty. Although the two could in principle be integrated, we were unable to find sufficient evidence in the literature to guide how they should be combined. It is possible to scale complexity by traffic measures, but this still raises unresolved questions about how to balance traffic against existing terms in the metric. How much should traffic contribute compared to left turns, right turns, or deviations from directness? Would traffic effects amplify or reduce the influence of these parameters as overall route complexity increases? Without empirical work to establish these relationships, integrating traffic into the current metric remains premature. Future research should therefore focus on quantifying the relative impact of traffic on each component before a combined measure can be justified. In future work, real-time traffic data or in-vehicle video could be used to evaluate the relationship between traffic and the complexity measure.

Finally, although the analysis was based on one year of data to mitigate seasonal effects and the region was limited to within the greater St. Louis area to ensure consistent traffic patterns and infrastructure, inherent variability in weather conditions and driving contexts across participants could not be fully controlled. With participants averaging one trip per week within the same city, some degree of inconsistency in driving conditions remains a potential source of variability.

#### 2) Data Resolution and Processing

The complexity measure is sensitive to data errors; for example, map matching has some limitations as the road networks change over time due to construction, which could impact the matched routes and turn identification. While map matching reduces the impact of the 30-second sampling rate of the device, it would be beneficial to get higher resolution data to account for cases where there are ambiguities in route options between two data-points. The turn detection similarly might count sharp curvatures in the road as a turn or miss wide turns.

#### 3) Weighting

We were unable to quantify the extent to which left turns impose greater cognitive load compared to right turns. Future work should focus on fine-tuning and optimizing the weight parameter to better capture and represent this difference. The weight parameter can be tailored based on the qualities of the road network or location. For example, high-traffic areas without traffic controls such as traffic lights might weight left turns higher than rural areas with low traffic.

#### 4) Unobserved Trip Context

Due to the observational nature of the study, we had no way of knowing whether participants used navigational aids such as a map, GPS, or passenger instructions to determine the route. Navigational aids can strongly influence the chosen route, particularly when in unfamiliar areas [46], [47]. We therefore recommend that future work collect more comprehensive data on whether navigation aids were used and how closely they were followed. This could be achieved through more active data collection methods, such as adding a post-trip survey or using in-vehicle video to observe whether navigation aids were in use.

We did not have self-reported data on the purpose of trips or participants’ final destinations. While we can observe where the vehicle stopped, we cannot infer whether the participant then walked to another nearby location, or the reason for the trip itself. For example, stopping at a strip mall could correspond to a medical appointment, grocery shopping, or meeting a friend. The type of destination likely influences the complexity of the route; for example, essential trips may favour more direct routes, while social or leisurely trips may involve local or familiar roads with higher turn density. Without this information, it is difficult to account for how the purpose of the trip may have contributed to the complexity of the route. Future work should consider collecting trip-purpose data, as differences in leisure versus essential trips may help explain variations in route selection. More contextual trip-level information such as destination type and navigation aid use can help to disentangle environmental constraints from cognitive factors in route complexity.

#### 5) Other Forms of Transport

An additional consideration is that this measure is specific to driving and cannot be directly extended to other modes of transportation, such as public transit or walking. With appropriate modifications, the metric could be adapted to active modes of transportation such as walking and cycling. For instance, when walking, left and right turns may impose similar demands, whereas for cycling, turns on a protected path may carry little added complexity, while turns across traffic could require higher cognitive load. By contrast, the metric does not translate to passive modes where individuals are not actively navigating, such as public transit or being driven by others. Our analysis also did not include multimodal trips (e.g., walking after parking) or travel by other means, as these data were not available. Nonetheless, mobility beyond driving remains an important consideration, since alternate forms of transportation provide additional insight into how individuals maintain independence and adapt their navigation strategies. Exploring these patterns across different modes could broaden the applicability of this measure in healthcare by encompassing a more comprehensive scope of mobility.

## V. Conclusion

This study demonstrates that preclinical AD may be associated with navigational behaviours in older adults as seen by the higher baseline route complexity and the reduction in complexity with advancing age. This study demonstrates the potential of our route complexity metric as a tool for analyzing spatial cognition and mobility in individuals with neurodegenerative conditions or cognitive impairment. Although challenges related to environmental variability and data processing exist, these findings suggest a promising direction for future research.

While not a diagnostic tool on its own, this metric illustrates that everyday navigational behaviours and decision making can provide signals that when combined with established biomarkers and other mobility measures, could help build a more comprehensive profile of cognitive health. This could help address the challenges currently faced by clinicians in confidently and accurately diagnosing and monitoring AD. Understanding behavioural changes associated with early signs of AD could therefore inform the work of clinicians and researchers, particularly in refining early detection and intervention strategies.

## Supplementary Materials

Supplementary Materials

## Appendix Map Matching

The sparse sampling rate of the trajectory data, with locations recorded every 30 seconds, failed to capture details of the driven path, including curves and turns. This led to an overly simplified representation of the route driven. To help solve this problem, we applied a map-matching algorithm to interpolate the on-road path travelled between each observed coordinate pair.

For each participant, a bidirectional graph representation of an Open Street Maps (OSM) road network was downloaded [31], where a graph is a set of nodes and edges. Nodes are stored as points representing intersections and ends of road segments, and edges define the geometry of the road network between nodes as a polyline. Each edge is associated with a start node and an end node, giving it direction. A bounding box was created, with each edge of the box being 0.2 degrees away from the home location, and the graph representation of the road networks within the bounding box was downloaded. If any trips were not within the bounding box, a 1.5 km buffer around the trajectory was computed, and the road networks within this buffer were added to the graph for that participant. Due to the large amount of data, a simplified graph was downloaded, meaning a limited number of nodes were included in the graph object, and they were only at intersections and corners.

The map-matching process used would find nodes near observed points to define the path, but not all routes start and end at a corner. To account for this, nodes were added to the graph near start and endpoints. To ensure the dataset remained small, the cluster centroids of the origins and destinations were used to identify start and end points, yielding a smaller number of nodes added. For each cluster centroid, the nearest edge was identified and projected onto the edge. The edge was divided into two edges at this point, and a new node was created between the new edges at the location of the projected node.

A fast map-matching algorithm [32] was employed using the modified graph and the trajectory data as inputs to estimate the on-road path taken for each trip. The algorithm includes a precomputing stage to speed up the map-matching process in which pairs of shortest paths in the road network were computed and stored in a searchable table. 8 candidate road segments were identified using k-nearest neighbours within a search radius of 0.003 degrees. A new graph was created with candidates as nodes and the precomputed shortest paths as edges. Using hidden Markov models, the likelihood of transitions between candidates based on GPS data was modelled, and a Viterbi algorithm was used to identify the most likely path through the candidate graph. The complete path was then reconstructed by combining the shortest paths between consecutive candidates.
