# Supplementary material for: Characterizing Navigational Changes in Preclinical Alzheimer’s Disease: A Route Complexity Metric Derived From Naturalistic Driving Data
Source: IEEE J Transl Eng Health Med. 2025 Oct 9;13:471–9. doi: 10.1109/JTEHM.2025.3619802 (PMC12599902; doi:10.1109/JTEHM.2025.3619802)
Supplement: Supplementary Materials [file supp1-3619802.doc]

# INTRODUCTION[[1]](#footnote-2)

# Supplementary Material: Comparison of Complexity of Routes Driven to Optimized Routes.

Here we provide an analysis supplementary to the main text comparing the complexity of actual and optimal routes for the same origin-destination pairs. In this supplement:

- The *actual* route refers to the path driven by the participant recorded with the GPS tracking device, after map matching (as described in Section II-B of the main article).

- The *optimal* route refers to a route optimized using contraction hierarchies, optimized for time without considering traffic.

By comparing actual to optimal complexity, we aim to benchmark observed behaviour against a theoretical ideal, in order to understand the potential of environmental impacts on available routes.

# Methodology

To manage computational load and avoid redundant routing, we first clustered participant start and end points to identify unique origin and destination locations. Because participants may not begin or end trips at exactly the same coordinates (due to parking, GPS quality, etc.), we grouped approximate origin/destination points using mean shift clustering [1]. The window size was set to 0.0008°, which is approximately 100 m on a sphere with a radius of 6378 km (the earth’s radius at the equator). This value was determined iteratively and validated visually.

Trips sharing the same origin and destination clusters were identified, and a request was sent to an open-source routing machine (OSRM) [2] to compute the fastest driving route, assuming no traffic. The OSRM request returned the optimized route trajectory along the road network, and the estimated distance travelled along the route. The turns were detected as described Section II-B of the main manuscript, and straight-line distance was also computed as described in the same section. We then applied the complexity measure (Equation (2) in the main text) to compute the complexity of both the actual and the optimal route as described in the main article.

We performed two statistical analyses to compare the actual to optimal routes:

### Actual vs. Optimal Complexity: We computed the average actual and optimal complexity values for each participant and then used a paired t-test to determine if the actual and optimal complexities differed.

### Actual to Optimal Ratio: We calculated the ratio of actual to optimal route complexity for each trip and then averaged these ratios for each participant. Because the data were not normally distributed, we used a Mann–Whitney U test to compare the mean actual-to-optimal complexity ratios between participants with preclinical Alzheimer’s disease and those in the control group.

# Results

In computing the optimal routes, the OSRM request failed for a small subset of trips (< 0.1%) which are therefore excluded from the results.

## Mean Actual vs Mean Optimal Complexity

The mean complexity of actual and optimal routes did not differ significantly across participants (t = −1.64, *p* = 0.10). The scatter plot of participant-level mean actual versus optimal complexity values (Fig. S1a) showed points distributed symmetrically around the identity line (*y* = *x*), suggesting no systematic bias toward higher or lower complexity. The distribution of mean differences ranged approximately from −0.3 to +0.3 and was approximately normal and centered near zero, indicating that, on average, participants drove routes with complexity similar to that of the computed optimal routes.

## Actual to Optimal Ratio

When comparing the median ratio of actual-to-optimal route complexity between participants with and without preclinical Alzheimer’s disease, we found no significant difference (U = 1349, *p* = 0.60). As shown in Fig. S2, both groups had median ratios slightly above 1 (Control: 1.097; Preclinical AD: 1.094), indicating that, on average, participants drove routes that were marginally less complex than the optimal benchmark. The distributions of ratios overlapped substantially between groups, although the range appeared larger for the preclinical AD group.

# Discussion

Participants generally drove routes that were similar to the optimal routes between their origins and destinations, as determined by OSRM. This alignment likely reflects the limited route options within the urban road network, where both actual and optimal routes may share substantial overlap and many trips—particularly short or arterial ones—are inherently simple. Consequently, observed route complexity may be influenced by environmental structure and route availability. For instance, some participants may have taken routes similar to the optimal path because few viable alternatives existed or because there were no compelling reasons to deviate, such as traffic congestion or complex intersections. As noted in Section IV-C of the main article, we could not determine whether navigation aids were used, which may also have contributed to the similarity between actual and optimal routes.

The approximately normal distribution of differences, centered near zero, suggests that while some participants occasionally took slightly more complex routes and others chose simpler ones, these deviations balanced out across the sample. However, because this supplementary analysis did not account for age or preclinical AD status, it cannot determine whether older participants or those with preclinical AD systematically deviated from optimal routes under similar environmental constraints.

As discussed in Section IV-C of the main article, environmental characteristics remain a key factor in route-planning behaviour. Although the study region is geographically limited, route complexity may still vary based on participants’ common destinations and residential locations. The optimal route provides a useful benchmark for regional comparisons; therefore, future work could benefit from analyses of long-term longitudinal data to examine whether the gap between actual and optimal route complexity diverges over time or with increasing age.

When comparing the actual-to-optimal complexity ratio between participants with and without preclinical Alzheimer’s disease, no statistically significant difference was observed. This suggests that preclinical AD may not influence the relative complexity of chosen routes within the road network compared to the optimal route. However, as noted in the main analysis, group differences in route complexity emerged only after adjusting for age; therefore, these supplementary findings do not alter the interpretation of the main results. Nonetheless, examining relative complexity longitudinally while accounting for age would provide valuable insights in future work.

# Conclusion

Taken together, these findings complement the discussion in the main article. They support the notion that the availability of alternative routes may influence route selection; however, behavioural factors cannot be ruled out given the absence of age- or group-specific adjustments.

References

[1] D. Comaniciu and P. Meer, “Mean shift: a robust approach toward feature space analysis,” *IEEE Trans. Pattern Anal. Mach. Intell.*, vol. 24, no. 5, pp. 603–619, May 2002, doi: 10.1109/34.1000236.

[2] D. Luxen and C. Vetter, “Real-time routing with OpenStreetMap data,” in *Proceedings of the 19th ACM SIGSPATIAL International Conference on Advances in Geographic Information Systems*, in GIS ’11. New York, NY, USA: Association for Computing Machinery, Nov. 2011, pp. 513–516. doi: 10.1145/2093973.2094062.

1. [↑](#footnote-ref-2)
